# Leukocyte TNFR1 and TNFR2 Expression Contributes to the Peripheral Immune Response in Cases with Ischemic Stroke

**DOI:** 10.3390/cells10040861

**Published:** 2021-04-09

**Authors:** Rikke B. Hansen, Cathrine C. H. Laursen, Niala Nawaz, Jonna S. Madsen, Helle H. Nielsen, Christina Kruuse, Arne Møller, Matilda Degn, Kate L. Lambertsen

**Affiliations:** 1 Department of Neurobiology Research, Institute of Molecular Medicine, University of Southern Denmark, 5000 Odense, Denmark; rikkebirkhansen@gmail.com (R.B.H.); laursencathrine@gmail.com (C.C.H.L.); ninaw19@student.sdu.dk (N.N.); Helle.Hvilsted.Nielsen@rsyd.dk (H.H.N.); 2 Department of Neurology, Odense University Hospital, 5000 Odense, Denmark; 3 Brain Research—Inter-Disciplinary Guided Excellence (BRIDGE), Department of Clinical Research, University of Southern Denmark, 5000 Odense, Denmark; 4 Department of Biochemistry and Immunology, Lillebaelt Hospital, University Hospital of Southern Denmark, 7100 Vejle, Denmark; Jonna.Skov.Madsen@rsyd.dk; 5 Department of Regional Health Research, University of Southern Denmark, 5000 Odense, Denmark; 6 Department of Clinical Medicine, University of Copenhagen, 2100 Copenhagen, Denmark; christina.kruuse@regionh.dk; 7 Department of Neurology, Herlev Gentofte Hospital, 2730 Herlev, Denmark; 8 Department of Nuclear Medicine and PET Center, Aarhus University Hospital, 8200 Aarhus, Denmark; arne@cfin.au.dk; 9 Institute of Clinical Medicine, Center of Functionally Integrative Neuroscience, 8000 Aarhus, Denmark; 10 Pediatric Oncology Laboratory, Department of Pediatrics and Adolescent Medicine, University Hospital Rigshospitalet, 2100 Copenhagen, Denmark; 11 OPEN—Open Patient data Explorative Network, Department of Clinical Research, Odense University Hospital, University of Southern Denmark, 5000 Odense, Denmark

**Keywords:** tumor necrosis factor receptor, inflammation, stroke, apoplexy, monocytes

## Abstract

Tumor necrosis factor receptor 1 and 2 (TNFR1 and TNFR2) have been found in brain parenchyma of stroke patients, and plasma levels are increased in the acute phase of stroke. We evaluated associations between TNFR1 and TNFR2 plasma levels and stroke severity, infarct size, and functional outcome. Furthermore, we examined cellular expression of TNFR1 and TNFR2 on leukocyte subpopulations to explore the origin of the increased receptor levels. Blood samples were taken from 33 acute ischemic stroke patients and 10 healthy controls. TNFR1 and TNFR2 plasma concentrations were measured and correlated against the Scandinavian Stroke Scale at admission, infarct volume, and the modified Rankin Scale score three months after stroke onset. Classical, intermediate, and non-classical monocytes as well as neutrophils were purified, and cellular expression of TNFR1 and TNFR2 was examined using flow cytometry. TNFR1 and TNFR2 plasma levels were both increased after ischemic stroke, but we found no correlation with patient outcome measurements. Compared to healthy controls, ischemic stroke patients had decreased non-classical monocyte and neutrophil populations expressing TNFR1 and increased neutrophils expressing TNFR2, and decreased non-classical populations co-expressing both TNFR1 and TNFR2. This study supports the hypothesis of an acute immunological response orchestrated by the peripheral immune system following an ischemic stroke. However, the origin of the increased TNFR1 and TNFR2 plasma levels could not be clearly linked to peripheral monocytes or neutrophils. Future studies are needed and will help clarify the potential role as treatment target.

## 1. Introduction

Ischemic stroke is the third leading cause of death and the leading cause of acquired disability in the Western world, and many stroke patients require assistance in daily life. An approved treatment strategy is recanalization therapy (thrombolysis and thrombectomy) in which the blood clot is dissolved or surgically removed, thereby improving patient outcome. Most stroke patients are not eligible for treatment, however, partly due to the short time window for treatment (4.5 h for thrombolysis and 24 h for thrombectomy) [1] or because of other contradicting factors [2]. Therefore, new treatment strategies are desperately needed to improve post-stroke outcome.

It is well established that ischemic stroke leads to an inflammatory response, and there is general agreement regarding the huge treatment potential in modulating the inflammatory response [1]. The pleiotropic cytokine tumor necrosis factor (TNF) is one of the most well-studied cytokines in relation to stroke and neuroinflammation, and it demonstrates both beneficial and detrimental effects (reviewed in [1]). TNF and its two receptors, TNFR1 and TNFR2, are upregulated in the human brain following stroke [1,3,4], and increased plasma TNF correlates with infarct volume in some studies [5] but not in others [6,7]; with stroke severity at admission in some studies [8,9,10] but not in others [11]; and with functional outcome in some studies [5,9], but not in others [12,13]. Furthermore, we [3] and others [14,15] demonstrated that TNFR1 and TNFR2 levels increase in the serum of ischemic stroke patients, but the role and source of increased blood TNFR1 and TNFR2 levels remain unclear. A particular role of TNF-TNFR1 signaling in the etiopathogenesis of stroke is suggested by genome-wide association studies that found a polymorphism in the TNF gene that increases susceptibility to ischemic stroke [16]. Furthermore, increased plasma TNFR1 and TNFR2 levels were associated with the risk of intracerebral hemorrhage [17], suggesting that TNF-mediated inflammation is associated with vascular changes preceding intracerebral hemorrhage.

However, the exact role exerted by TNF and its two receptors in the context of ischemic stroke is still controversial. TNF exists in two biologically active forms, transmembrane TNF (tmTNF) and soluble TNF (solTNF), with solTNF having the highest affinity for TNFR1 and tmTNF the highest affinity for TNFR2 [18,19,20]. In most neurological diseases, such as multiple sclerosis, Alzheimer’s disease, and traumatic brain injury, solTNF-TNR1 signaling appears to be detrimental, whereas tmTNF-TNFR2 signaling appears to be beneficial (reviewed in [21,22,23,24]). In ischemic stroke, however, tmTNF-TNFR1-signaling appears to be protective [25,26,27,28,29]. Whereas TNFR1 is ubiquitously expressed on most cell types, TNFR2 is mainly expressed on immune cells. Given that ischemic stroke is an acute cerebrovascular event with delayed infiltration of immune cells, it is possible that TNFR1 signaling is crucial for neuronal survival.

Neutrophils and monocytes infiltrate the brain parenchyma after stroke onset, with monocytes being recruited preferentially through the chemokine (C-C motif) ligand 2/C-C chemokine receptor type 2 (CCL2/CCR2) axis [30]. In mice, CCR2 is mainly expressed on a subset of monocytes expressing high levels of Ly6C, but low levels of CX3C chemokine receptor 1 (CX3CR1). These are often referred to as either Ly6C^high^ or inflammatory monocytes, as many Ly6C^high^ monocytes are recruited to inflamed tissue in a CCR2-dependent manner, where they then produce Th1-type pro-inflammatory cytokines [31]. A small proportion (~18%) of Ly6C^low^ monocytes also expresses cell-surface CCR2 [32]. In mice, CCR2^+^Ly6C^high^ monocytes appear to be the predominant monocyte subtype to infiltrate the ischemic brain after a permanent middle cerebral artery occlusion [33]. In experimental permanent stroke models, CCL2 deficiency leads to a decrease in the number of monocytes recruited to the ischemic lesion and as a consequence the mice develop smaller infarct volumes post-stroke [30]. Furthermore, compared to wildtype mice, CCR2-deficient mice develop smaller infarcts, reduced blood–brain barrier (BBB) permeability, reduced expression of inflammatory cytokines, and reduced infiltration not only of monocytes but also of neutrophils after ischemia-reperfusion [34]. This indicates that infiltration of peripheral immune cells through the CCL2/CCR2 axis contributes to the neuroinflammation present after experimentally induced stroke.

In humans, three monocyte subsets have been described: classical CD14^++^CD16^−^ (analogous to murine Ly6C^high^CCR2^high^CX3CR1^low^), intermediate CD14^+^CD16^+^, and non-classical CD14^+^CD16^++^ (analogous to murine Ly6C^low^CCR2^low^CX3CR1^high^) monocytes, with the intermediate subset proposed as a monocyte in transition from a classical to a non-classical monocyte [35]. In stroke patients, an increased proportion of circulating classical monocytes is associated with poor outcome, higher mortality, and early clinical worsening [36]. Intermediate monocytes are inversely related to mortality, and non-classical monocytes are inversely related to poor outcome and infarction size [36]. These results suggest that the classical monocytes convey harmful effects after stroke and that intermediate and non-classical monocytes are beneficial with a phenotype that could promote tissue repair [36]. Selective targeting of chemokine or cytokine receptors may allow manipulation of specific immune cell types that enter the brain after stroke. However, whether acute stroke also results in changes in monocyte subset CCR2, TNFR1, and TNFR2 expression remains to be elucidated.

As ischemic stroke leads to acute breakdown of the BBB [37], partly caused by increased vascular endothelial growth factor (VEGF)-A levels [38,39], brain proteins quickly leak into the blood. We [40] and others [41,42,43,44,45] previously showed that brain-derived neuronal and glial markers, such as neurofilament light-chain (NF-L) and glial fibrillary acidic protein (GFAP), can be predictive biomarkers for stroke severity on admission and functional outcome. In addition, brain-derived inflammatory markers, such as cerebrospinal fluid levels of interleukin (IL)-6, have been shown to correlate with infarct volume and functional outcome [46,47].

In this study, we compared blood TNF, TNFR1, TNFR2, CCL2, and CCR2 levels in stroke patients and healthy controls and investigated the association of TNFR1, TNFR2, CCL2, and CCR2 levels to stroke severity, infarct size, and functional outcome at 90 days. We also evaluated an association between TNFR1 or TNFR2 levels and blood NF-L, GFAP, IL-6, or VEGF-A levels. In addition, we evaluated post-stroke changes in monocyte subsets and neutrophils, along with their surface expression of CCR2, TNFR1, and TNFR2.

## 2. Materials and Methods

### 2.1. Ethics Approval and Consent to Participate

Patient recruitment was conducted in accordance with the Helsinki II declarations. Each patient and healthy control was given both written and oral information about the study and the extent of their involvement. All participants provided written informed consent prior to study participation, and a copy of this was made available. Participants were informed of their right to retract consent at any given time point, which no one did in this setting.

The study was approved by the National Committee on Health Research Ethics in Denmark (Project-ID: S-20160152G) and has been registered with the Danish Data Protection Agency (J. No. 16/34165).

### 2.2. Participants

Patients were included consecutively from June 19th to November 29th, 2019 and comprised patients admitted to the Department of Neurology, Odense University Hospital, Denmark who presented with stroke symptoms, were admitted within 48 h of symptom onset, and were over 18 years of age. Patients receiving thrombolysis and/or thrombectomy were also included. To ensure proper informed written consent, only patients who read and understood Danish and patients without severe aphasia were included. Exclusion criteria were cerebral space-occupying lesions, hemorrhage, sinus venous thrombosis, and pregnancy. Patients who were later discharged with a diagnosis other than ischemic stroke (ICD-10 code I63) were excluded (and the patients were informed). In total, 38 patients meet the inclusion criteria. Healthy relatives were recruited as controls (*n* = 10).

For the infarct volumetric and correlation analyses, an additional 26 ischemic stroke patients, 11 for TNFR1 and TNFR2 analyses (Table 1) and 26 for CCL2 and CCR2 analyses (Table S1), from previously published papers [3,40] were included for data on Scandinavian Stroke Scale (SSS), modified Rankin Scale (mRS), time passed from stroke onset, and TNFR1 and TNFR2 levels (see below). These patients were recruited from October 2017 to February 2018 using the same criteria described as described above. In addition, 7 controls from previously published papers [3,40] were included for CCL2 and CCR2 analyses. Comparable characteristics of the groups of patients used for characterization of serum CCL2 and CCR2 levels are presented in Table S1.

All data are hosted at Open Patient data Explorative Network (OPEN; https://open.rsyd.dk/) and requests to access datasets should be directed to klambertsen@health.sdu.dk.

### 2.3. Patient Demographics

All patients with suspected stroke had a standard non-contrast head computed tomography (CT) scan performed on admission. Seventeen patients also had a follow-up MRI scan. SSS [48] was used to assess stroke severity on admission; the score range is 0–58, where a low score indicates greater stroke severity. Data were accessed from patient files on sex, age, smoking status (current, former, or never smoker), drinking habits (higher or lower intake than recommended by the Danish health authorities), prescribed anti-inflammatory medication (NSAIDs, tropical, and systemic glucocorticoids), differential leukocyte count at admission, and diagnosis at discharge. The mRS was used to estimate the degree of disability and dependence after stroke [49]. This assessment was made by telephone interview three months after stroke onset.

Healthy controls were asked for information on age, smoking status, drinking habits, medicine consumption, and comorbidity.

### 2.4. Sample Collection and Handling

Blood was collected by vein puncture in 4 and 10 mL EDTA tubes (BD Biosciences, San Jose, CA, USA) or 4 mL vacutainer tubes for ELISA and chemiluminescence analysis or leukocyte purification followed by flow cytometry analysis, respectively. The timepoint of the first symptom stated in patient records was used to estimate the time passed from stroke onset to blood sampling. In the case of wake-up stroke, the time for wake-up was chosen.

Within 30–60 min after sampling, blood was centrifuged at 2000 *g* for 10 min at room temperature, and plasma or serum was aliquoted and stored at −80 °C until analysis.

### 2.5. Chemiluminescence Analysis

Plasma concentrations of TNF were measured using V-PLEX Human Proinflammatory Panel 1 and plasma concentrations of TNFR1 and TNFR2 were measured using Human TNFR-I ultra-sensitive kit and Human TNFR-II ultra-sensitive kit. Interleukin (IL)-6 and vascular endothelial growth factor (VEGF-A) were measured using the V-Plex Human IL-6 kit and V-Plex Human VEGF kit (all from Mesoscale Discovery, Rockville, MD, USA). Serum concentrations of CCL2 were measured using a V-PLEX human MCP-1 kit (Mesoscale Discovery). Samples were diluted in Diluent-41 and analyzed in duplicate according to the manufacturer’s instructions. Analysis was performed on a SECTOR Imager 6000 plate reader and MSD Discovery Workbench software was used for analysis (Mesoscale Discovery). Sample replicates with coefficient of variation (CV) values >25% in individual analyses were excluded. None of the measurements were below the lower level of detection.

### 2.6. Simoa Analysis

Serum concentrations of neurofilament light chain (NF-L) and glial fibrillary acidic protein (GFAP) in controls and ischemic stroke patients were analyzed at the Department of Biochemistry and Immunology, Lillebaelt Hospital, Vejle being accredited by Danish Accreditation Fund (DANAK) according to the ISO 15189:2012 standard that specifies requirements for quality and competence in medical laboratories. Both NF-L and GFAP were measured blinded to clinical data by single molecule array technology (Simoa, HD-X Analyzer (Quanterix, Billerica, MA, USA)) [40], using the commercially available 2-Plex assay for the quantitative determination of NF-L and GFAP in human serum (Quanterix). Quality control was performed using five internal controls in each run. Internal controls were prepared from commercially available control material provided by the manufacturer in addition to an in-house prepared serum pool. The in-house serum pool was used as an internal control and included in each run for evaluating and monitoring assay performance over time. The total analytical variation for the included controls were 10–16% total analytical CV for NF-L and 8–14% total analytical CV for GFAP. Lower limits of detection for NF-L and GFAP were 0.038 and 0.211 pg/mL, respectively, whereas the lower levels of quantification were 0.174 and 0.686 pg/mL, respectively.

### 2.7. ELISA Analysis

Serum concentrations of human CCR2 were measured using a double-antibody sandwich ELISA (Cloud-Clone Corp) according to the manufacturer’s instructions. Samples were run in duplicate, and analysis performed on an ELISA Versa Max reading and data analyzed using SoftMax Pro 7.0.2 software (ThermoFisher, Waltham, MA, USA). A CV of <20% was accepted.

### 2.8. Leukocyte Isolation

Blood samples were kept at 4 °C for a maximum of 24 h. For leukocyte isolation, whole blood was diluted 1:3 in phosphate-buffered saline (PBS) (pH 7.45, ThermoFisher), placed on top of Leuko Spin Medium (PluriSelect, Leipzig, Germany), and centrifuged at 1200× *g* for 30 min at room temperature (brakes off). The leukocyte fraction was washed three times in PBS (300× *g* for 10 min at room temperature) followed by the addition of freezing medium (40% fetal calf serum and 20% dimethyl sulfoxide (Sigma-Aldrich, Søborg, Denmark) in RPMI 1640 (ThermoFisher)). Samples were frozen to −80 °C at a rate of −1 °C/min using a CoolCell SV2 freezing container (BioCision, Larkspur, CA, USA) until further analysis.

### 2.9. Flow Cytometry

Prior to flow cytometry analysis, leukocyte samples were thawed and rapidly diluted in RPMI. Erythrocytes were lysed for 8 min using erythrolyse solution (Bio-Rad, Copenhagen, Denmark). The pellets were resuspended in PBS containing 1% human serum (Sigma-Aldrich), and leukocytes were counted (NucleoCounter NC-200, Chemometec, Lillerød, Denmark) to ensure 1,500,000 cells for analysis. Unspecific binding was blocked using 10% human serum (Sigma-Aldrich), True-Stain Monocyte Blocker (BioLegend, San Diego, CA, USA), and mouse IgG2a negative control (Agilent, Glostrup, Denmark) diluted in PBS for 15 min.

The cells were washed and stained for extracellular surface markers: CD16-FITC (clone 3G8, BioLegend), CD14-PerCP-Cy5.5 (clone M5E2, BD Biosciences), HLA-DR-APC-Cy7 (clone L243, BD Biosciences), CD13-BV480 (clone L138, BD Biosciences), CCR2-BV421 (clone 48607, BD Biosciences), CD3-PE-Cy7 (clone SK7, BD Biosciences), CD20-PE-Cy7 (clone 2H7, BD Biosciences), TNFR1-PE (clone W15099A, BioLegend), and TNFR2-Alexa Flour 647 (clone 22235, R&D Systems, Minneapolis, MN, USA) for 30 min at 4 °C in the dark.

Next, the cells were washed and fixed using Cytofix/Cytoperm Solution (BD Biosciences) for 20 min at 4 °C in the dark. After a final wash, the cells were resuspended in PBS containing 1% human serum and kept in the dark at 4 °C overnight until analysis.

The concentration used of each antibody was optimized by titration analysis, and fluorescence minus one (FMO) controls were used to set the gates for analysis. To correct for unwanted, unspecific binding, the corresponding isotype controls were used: mouse IgG2_a_-Alexa Flour 647 (clone 20102, R&D Systems) for TNFR2; mouse IgG2_a,κ_-PE (clone MOPC-173, Biolegend) for TNFR1; and mouse IgG1_κ_-FITC (clone MOPC-21, Biolegend) for CD16. Flow cytometry analysis was performed on 1,000,000 cells per sample and analyzed using a FACSverse multicolor flow cytometer equipped with FACSuite software (BD Biosciences). The mean fluorescence intensity (MFI) was calculated as the geometric mean of each population in the CCR2, TNFR1, and TNFR2 positive gates, respectively.

### 2.10. Infarct Volume Estimation

MRI scans were used to estimate the total infarct volume (IFV). When available, both diffusion-weighted imaging (DWI) and apparent diffusion coefficient (ADC) images were used to identify subacute lesions. Infarct volumes were estimated on DWI scans using a counting grid placed at random and images were enlarged 8× to ensure better accuracy. All points within the infarct were counted and the volume was calculated using Calvalieri’s principle:

V=t∗a(p)∗ΣP

with V being the infarct volume of each lesion (mm^3^), t the distance between each section of the scan (mm), a(p) the constant area between the points counted (mm^2^), and ΣP the number of points counted within the infarct [50]. In MRI scans containing multiple lesions, all lesions were estimated and calculated separately resulting in a total volume used for analysis.

### 2.11. Statistical Analysis

Fisher’s exact test was used to test differences in sex and pre-hospital anti-inflammatory treatment between stroke patients and healthy controls; chi-square test was used to compare smoking status and drinking habits; and Mann–Whitney test was used to compare age. D’Agostino and Person omnibus normality test and Shapiro–Wilk normality test were used to assess normal distribution. As most of the data were not normally distributed, all data were analyzed as non-parametric data. For comparison between groups, Mann–Whitney U test was used, and correlation analyses were completed using Spearman correlation analysis to account for non-linear relationships between covariates. SSS and mRS were treated as continuous variables, and controls were assumed to score 0 on mRS and 58 on SSS.

Data are presented as percentages, median with interquartile range; (IQR 25–75%), 2.5–97.5% percentiles, or mean with 95% confidence interval (CI), and *p* ≤ 0.05 was considered statistically significant. All statistical analyses were performed using Prism 6 software for Mac (GraphPad, San Diego, CA, USA).

## 3. Results

After excluding five patients due to a different discharge diagnosis, the study cohort consisted of 33 ischemic stroke patients and 10 healthy individuals. The patient group had significantly more males (61%) than control group (20%; *p* = 0.03) (Table 1), and the patients were significantly older (median 73 years, average 71 years, 95% CI: 66–75 years) than the healthy controls (median 59, 95% CI: 54–69 years; *p* = 0.02) (Table 1). There were no differences between the two groups in smoking status, drinking habits, or anti-inflammatory treatment (Table 1). Blood samples were taken 1.6–64.9 h after stroke onset, with an average of 24.4 h (Table 1). In two cases of wake-up stroke, the time of first symptom was unknown, and the time for wake-up was used.

Table 1 also shows the characteristics of the 11 additional patients from two recent studies [3,40] used for TNFR1 and TNFR2 correlation analyses.

### 3.1. Distribution of Peripheral Immune Cells

**Figure 1:**
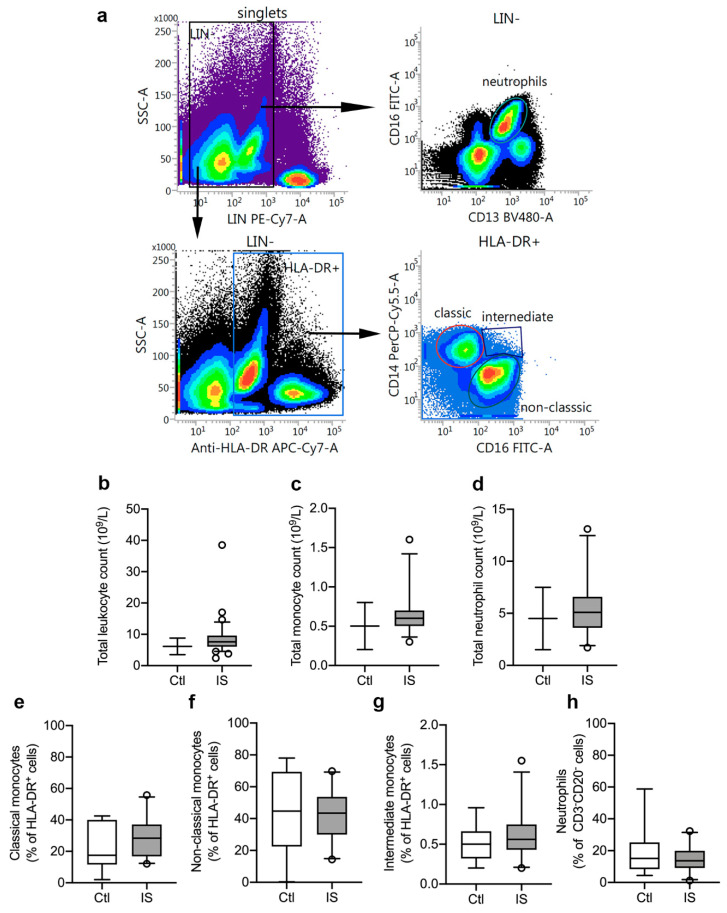
Characterization of leukocyte populations in ischemic stroke (IS) patients. (**a**) Flow cytometry gating strategy for blood monocyte subsets and neutrophils. Peripheral blood monocytes were identified as LIN^−^ (CD3 and CD20) HLA-DR^+^ cells and neutrophils as LIN^−^ (CD3 and CD20) CD16^+^CD13^+^ cells (yellow gated population). The monocyte population comprised classical monocytes (CD14^++^CD16^−^: red gated population), intermediate monocytes (CD14^++^CD16^+^: blue gated population), and non-classical monocytes (CD14^+^CD16^++^: green gated population). (**b**–**d**) Total leukocyte count (**b**), total monocyte count (**c**), and total neutrophil count (**d**) in IS patients (*n* = 33) compared to the normal population range (control, Ctl). (**e**–**h**) Quantification of classical monocytes (**e**), non-classical monocytes (**f**), intermediate monocytes (**g**), and neutrophils (**h**) in IS (*n* = 23) and healthy Ctl (*n* = 8). Line: median. Box: 25–75% interquartile range. Whiskers: 5–95% percentile.

We initially estimated leukocyte cell populations using flow cytometry (Figure 1a) and leukocyte differential count (Figure 1b–d). Total leukocyte differential count was increased in ischemic stroke patients (mean: 8.9 × 10^9^/L) compared to the normal population reference range: 3.5–8.8 × 10^9^/L (Figure 1b and Table 2). The count of thrombocytes, lymphocytes, monocytes, and neutrophils were all within the IQR reference ranges, although monocyte and neutrophil counts were in the upper end of the IQR range (monocytes: 0.5–0.7 × 10^9^/L, reference range: 0.2–0.8 × 10^9^/L; and neutrophils: 3.6–6.6 × 10^9^/L, reference range: 1.5–7.5 × 10^9^/L (Figure 1c,d and Table 2).

Using flow cytometry, we estimated the leukocyte cell populations (%) in ischemic stroke patients (*n* = 23) and healthy controls (*n* = 8). CD3^−^CD20^−^HLA-DR^+^ cell populations were similar in controls [31.2% (23.8–35.1)] and ischemic stroke patients [30.6% (23.9–37.1)] (U = 88, *p* = 0.88). HLA-DR^+^ cells were then further gated into CD14^++^CD16^−^ classical monocytes (Figure 1e), CD14^+^CD16^++^ non-classical monocytes (Figure 1f), and CD14^++^CD16^+^ intermediate monocytes (Figure 1g). We observed no significant differences between controls and patients for classical monocytes (controls (Ctl): 17.5% (11.7–40.1) and ischemic stroke (IS): 28.4% (16.9–37.1), U = 70, *p* = 0.34), non-classical monocytes (Ctl: 44.7% (22.4–69.4) and IS: 43.4% (29.9–53.7), U = 82, *p* = 0.67), and intermediate monocytes [Ctl: 0.50% (0.32–0.66) and IS: 0.56% (0.43–0.75), U = 75.5, *p* = 0.47]. Among CD3^−^CD20^−^ leukocytes (Figure 1h), we found no significant difference (U = 86, *p* = 0.81) in the neutrophil populations of Ctl [15.1% (8.3–25.1)] and IS [13.7% (9.1–19.9)].

### 3.2. CCR2 and CCL2 Blood Levels

**Figure 2:**
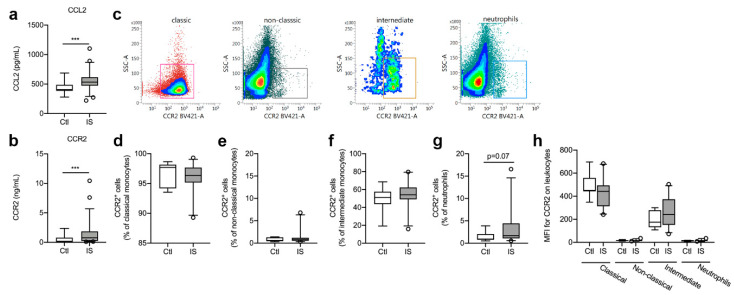
Characterization of CCR2 cell populations in ischemic stroke (IS) patients. (**a**) Electrochemiluminescence analysis of CCL2 plasma concentrations in IS patients (*n* = 58) and healthy controls (Ctl, *n* = 16), demonstrating significantly increased CCL2 plasma levels in IS patients compared to healthy Ctl. (**b**) ELISA analysis of CCR2 plasma concentrations in IS patients (*n* = 58) and healthy Ctl (*n* = 16), demonstrating significantly increased CCR2 plasma levels in IS patients compared to healthy Ctl. (**c**) Flow cytometry analysis of CCR2 expression on classical monocytes (red gate), non-classical monocytes (black gate), intermediate monocytes (orange gate), and neutrophils (blue gate) in IS. (**d**–**g**) Quantification of the percentage of CCR2^+^ classical monocytes (**d**), CCR2^+^ non-classical monocytes (**e**), CCR2^+^ intermediate monocytes (**f**), and CCR2^+^ neutrophils (**g**) in IS compared to healthy Ctl. (**h**) Mean fluorescence intensity (MFI) of CCR2 on classical, non-classical, and intermediate monocytes and on neutrophils in IS (*n* = 23) and healthy Ctl (*n* = 8). Line: median. Box: 25–75% interquartile range. Whiskers: 5–95% percentile. *** *p* < 0.001.

We found that serum CCL2 levels were significantly increased in ischemic stroke patients compared to healthy controls (Figure 2a, U = 185, *p* = 0.0001).

No correlation was found between CCL2 and time to blood sample (Spearman’s rho = 0.07, *p* = 0.62), SSS (Spearman’s rho = −0.21. *p* = 0.09), IFV (Spearman’s rho = −0.06, *p* = 0.83), or mRS (Spearman’s rho = 0.12, *p* = 0.43).

In addition, serum CCR2 levels were significantly increased in ischemic stroke patients compared to healthy controls (Figure 2b, U = 218, *p* = 0.0009) and CCR2 levels correlated significantly with time to blood sample (Spearman’s rho = −0.28, *p* = 0.04), SSS (Spearman’s rho = −0.26, *p* = 0.04), and mRS (Spearman’s rho = 0.31, *p* = 0.045). No correlation was found between CCR2 and IFV (Spearman’s rho = 0.32, *p* = 0.21).

### 3.3. CCR2^+^ Cell Populations were Comparable in Healthy Controls and Ischemic Stroke Patients

Classical, non-classical, and intermediate monocytes along with neutrophils were gated for CCR2 expression (Figure 2c). The population of CCR2^+^ classical monocytes (Figure 2d) was comparable in controls [97.7% (94.2–98.2)] and ischemic stroke patients [96.3% (95.2–97.7), U = 0.24, *p* = 0.24]. CCR2^+^ non-classical monocyte cell populations (Figure 2e) were also comparable in controls [0.60% (0.42–1.31)] and ischemic stroke patients [0.78% (0.52–1.19), U = 75, *p* = 0.46], as were CCR2^+^ intermediate monocyte cell populations (Figure 2f) [Ctl: 51.2% (43.8–57.6) and IS: 54.2% (49.0–62.4), U = 65, *p* = 0.24]. When comparing CCR2^+^ neutrophil cell populations (Figure 2g), we found a trend towards more CCR2^+^ neutrophil populations in ischemic stroke patients [1.68% (1.09–4.44)] compared with controls [0.93% (0.78–2.04), U = 52.5, *p* = 0.07]. MFI for CCR2 on classical monocytes was high, intermediate monocytes had a dim expression of CCR2, whereas non-classical monocytes and neutrophils were CCR2^−^ (Figure 2h). We observed no differences in MFI for CCR2 on classical monocytes [Ctl: 451 (442–559) and IS: 442 (313–494), U = 61.5, *p* = 0.17] or intermediate monocytes [Ctl: 175 (131–279) and IS: 240 (151–373), U = 58.5, *p* = 0.13] (Figure 2h).

### 3.4. Plasma Levels of TNFR1 and TNFR2 were Significantly Increased in Ischemic Stroke Patients

**Figure 3:**
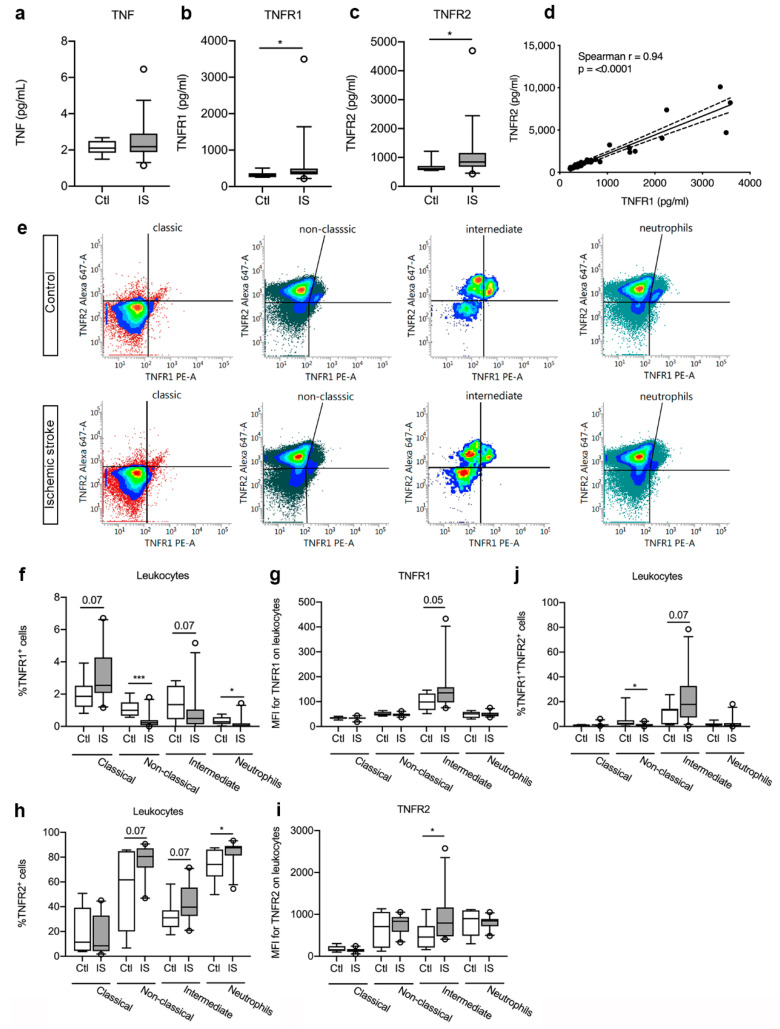
Characterization of TNFR1 and TNFR2 levels and cellular expression in ischemic stroke (IS) patients. (**a**–**c**) Electrochemiluminescence analysis of TNF (**a**), TNFR1 (**b**), and TNFR2 (**c**) plasma concentrations in IS patients (*n* = 33) and healthy controls (Ctl, *n* = 10), demonstrating significantly increased TNFR1 and TNFR2 plasma levels in IS patients compared to healthy Ctl. (**d**) Correlation analysis of TNFR1 and TNFR2 plasma levels in IS patients (*n* = 44), demonstrating a significant positive correlation between TNFR1 and TNFR2 levels post-stroke (*p* < 0.0001). Line: linear regression through the data points. Dotted line: 95% confidence interval of linear regression. (**e**) Flow cytometry analysis of TNFR1 and TNFR2 expression on classical monocytes, non-classical monocytes, intermediate monocytes, and neutrophils in IS patients (*n* = 23) and healthy Ctl (*n* = 8). (**f**) Quantification of the percentage of TNFR1^+^ monocyte subsets and neutrophils in IS patients and healthy Ctl. (**g**) Mean fluorescence intensity (MFI) of TNFR1 on classical, non-classical, and intermediate monocytes and on neutrophils in IS and healthy Ctl. (**h**) Quantification of the percentage of TNFR2^+^ monocyte subsets and neutrophils in IS patients and healthy Ctl. (**i**) MFI of TNFR2 on classical, non-classical, and intermediate monocytes and on neutrophils in IS and healthy Ctl. (**j**) Quantification of the percentage of TNFR1^+^TNFR2^+^ monocyte subsets and neutrophils in IS patients and healthy Ctl. Line: median. Box: 25–75% interquartile range. Whiskers: 5–95% percentile. TNF, tumor necrosis factor; TNFR, tumor necrosis factor receptor. * *p* < 0.05, *** *p* < 0.001.

Plasma TNF levels (Figure 3a) were comparable in controls and ischemic stroke patients (*p* = 0.50, Table 3). In contrast, plasma TNFR1 levels (Figure 3b) were significantly increased in ischemic stroke patients compared to healthy controls (*p* = 0.04, Table 3). Plasma TNFR2 levels (Figure 3c) were also significantly increased in ischemic stroke patients compared to controls (*p* = 0.03, Table 3). To increase the number of individual measurements for correlation analyses, we then included TNFR1 and TNFR2 levels measured in ischemic stroke patients under the same conditions in a recent previous study [40] and estimated whether TNFR1 and TNFR2 levels correlated post-stroke. Interestingly, we found that TNFR1 and TNFR2 levels were positively correlated to each other in ischemic stroke patients (Spearman’s rho = 0.94, *p* < 000.1) (Figure 3d).

### 3.5. TNFR Expression on Peripheral Immune Cells

As plasma TNFR1 and TNFR2 levels increased after ischemic stroke, we aimed to locate the origin of this response and used flow cytometry to estimate TNFR1 and TNFR2 expression on monocyte subpopulations and neutrophils in controls and ischemic stroke patients (Figure 3e). We found a trend towards an increase in the TNFR1^+^ classical monocyte cell population [Ctl: 1.87% (1.21–2.52) and IS: 2.54% (2.07–4.27), U = 51.5, *p* = 0.07] in ischemic stroke patients compared to controls, whereas the TNFR1^+^ intermediate cell population showed a trend towards a decrease [Ctl: 1.35% (0.44–2.52) and IS: 0.49% (0.13–1.04), U = 52, *p* = 0.07] (Figure 3f). The TNFR1^+^ non-classical monocyte cells [Ctl: 1.00% (0.66–1.49) and IS: 0.23% (0.07–0.39), U = 15, *p* < 0.001] and the TNFR1^+^ neutrophil cells [Ctl: 0.28% (0.15–0.57) and IS: 0.05% (0.01–0.19), U = 39.5, *p* = 0.02] cell populations significantly decreased in ischemic stroke patients (Figure 3f). MFI for TNFR1 was low on classical and non-classical monocytes, as well as on neutrophils (Figure 3g). Despite the trend towards decreased TNFR1^+^ intermediate monocyte populations in ischemic stroke patients compared to healthy controls (Figure 3f), MFI for TNFR1 on the cells increased in ischemic stroke patients (U = 49, *p* = 0.05) (Figure 3g), suggesting upregulation of TNFR1 on this cell population.

The TNFR2^+^ classical monocyte population was comparable in controls [11.41% (4.39–39.18)] and ischemic stroke patients [8.49% (4.10–32.86), U = 79.5, *p* = 0.59] (Figure 3h), but the TNFR2^+^ non-classical monocyte cells [Ctl: 61.72% (20.02–84.81) and IS: 80.50% (71.51–87.15), U = 52, *p* = 0.07] and intermediate monocyte cells [Ctl: 31.02% (23.52–37.16) and IS: 39.56% (32.45–55.41), U = 52, *p* = 0.07] both showed a trend towards an increase in ischemic stroke patients compared to controls (Figure 3h). The TNFR2^+^ neutrophil cell population was significantly increased in ischemic stroke patients [87.53% (81.36–89.17)] compared to healthy controls [74.17% (64.41–86.10), U = 39, *p* < 0.05] (Figure 3h). MFI for TNFR2 on classical monocytes, non-classical monocytes, and neutrophils were comparable in controls and ischemic stroke patients, whereas MFI for TNFR2 was significantly increased on intermediate monocytes in ischemic stroke patients [Ctl: 459 (210–722) and IS: 793 (475–1165), U = 43, *p* < 0.05] (Figure 3i).

Finally, we observed a significant decrease in TNFR1^+^TNFR2^+^ non-classical monocytes in ischemic stroke patients compared to controls [Ctl: 2.48% (1.41–5.04) and IS: 0.95% (0.25] 1.92), U = 41, *p* < 0.05], whereas TNFR1^+^TNFR2^+^ intermediate monocytes showed a trend towards an increase [Ctl: 13.24% (1.80–14.23) and IS: 17.80% (7.24–32.67), U = 52, *p* = 0.07] (Figure 3j). TNFR1^+^TNFR2^+^ classical monocytes [Ctl: 1.16% (0.13–1.39) and IS: 1.30% (0.84–1.74), U = 64, *p* = 0.22] and TNFR1^+^TNFR2^+^ neutrophil [Ctl: 1.19% (0.71–2.45) and IS: 0.75% (0.23–2.71), U = 74, *p* = 0.44] cell populations were comparable in the two groups (Figure 3j).

### 3.6. TNFR1 and TNFR2 Expression on CCR2^+^ Leukocytes was Unchanged in Acute Ischemic Stroke in Humans

**Figure 4:**
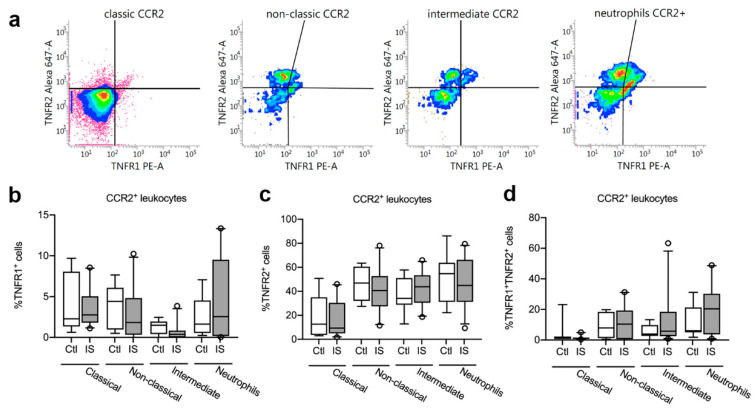
Characterization of TNFR1 and TNFR2 expression on CCR2^+^ monocytes and neutrophils. (**a**) Flow cytometry analysis of TNFR1 and TNFR2 expression on CCR2^+^ classical, non-classical, and intermediate monocytes and on neutrophils in ischemic stroke (IS) patients (*n* = 23) and healthy controls (Ctl, *n* = 8). (**b**) Quantification of the percentage of TNFR1^+^ CCR2^+^ monocyte subsets and neutrophils in IS and healthy Ctl. (**c**) Quantification of the percentage of TNFR2^+^ CCR2^+^ monocyte subsets and neutrophils in IS and healthy Ctl. (**d**) Quantification of the percentage of TNFR1^+^TNFR2^+^ CCR2^+^ monocyte subsets and neutrophils in IS and healthy Ctl. Line: median. Box: 25–75% interquartile range. Whiskers: 5–95% percentile. TNFR, tumor necrosis factor receptor.

We next gated for TNFR1 and TNFR2 expression on CCR2^+^ classical, non-classical, and intermediate monocytes and CCR2^+^ neutrophils (Figure 4a). We observed no differences in the percentages of TNFR1^+^ CCR2^+^ classical monocytes, non-classical monocytes, intermediate monocytes, or TNFR1^+^ CCR2^+^ neutrophils between healthy controls and ischemic stroke patients (Figure 4b and Table 4).

We also observed no differences in the percentages of TNFR2^+^ CCR2^+^ classical monocytes, non-classical monocytes, intermediate monocytes, or TNFR2^+^ CCR2^+^ neutrophils between healthy controls and ischemic stroke patients (Figure 4c and Table 4).

Finally, there were no differences in the percentages of TNFR1^+^TNFR2^+^ CCR2^+^ classical monocytes, non-classical monocytes, intermediate monocytes, or TNFR1^+^TNFR2^+^ CCR2^+^ neutrophils between healthy controls and ischemic stroke patients (Figure 4d and Table 4).

Interestingly, plasma CCL2 levels positively correlated with plasma TNFR1 (Spearman’s rho = 0.34, *p* = 0.046) and TNFR2 (Spearman’s rho = 0.39, *p* = 0.02) but no association was found for CCR2 to TNFR1 (Spearman’s rho = −0.24, *p* = 0.17) or TNFR2 (Spearman’s rho = 0.13, *p* = 0.44).

### 3.7. Serum NF-L and GFAP Levels Are Significantly Increased in Ischemic Stroke Patients

**Figure 5:**
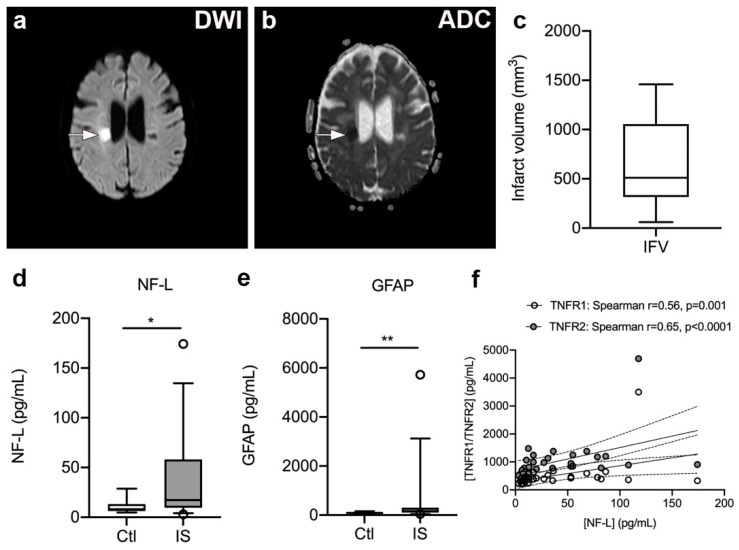
Infarct volumetric analysis in ischemic stroke patients. (**a**,**b**) Diffusion-weighted imaging (DWI) (a) and apparent diffusion coefficient (ADC) (**b**) images of a subacute ischemic infarct in a 58-year-old woman with a subcortical lacunar infarct in the right hemisphere. (**c**) Volumetric analysis of the size of the infarct obtained on MRI images derived from ischemic stroke patients (*n* = 17). (**d**,**e**) Neurofilament light chain (NF-L) (**d**) and glial fibrillary acidic protein (GFAP) (**e**) concentrations increased significantly in the serum of ischemic stroke (IS) patients (*n* = 33) compared to controls (Ctl) (*n* = 10). Line: median. Box: 25–75% interquartile range. Whiskers: 5–95% percentile. * *p* < 0.05, ** *p* < 0.01. (**f**) Correlation analysis of serum NF-L levels and plasma TNFR1 or TNFR2 levels in IS patients (*n* = 33) demonstrating a significant positive correlation between NF-L and TNFR1 or TNFR2 levels post-stroke. Line: linear regression through the data points. Dotted lines: 95% confidence intervals of linear regression.

To estimate infarct volumes, we localized infarcted brain tissue on DWI (Figure 5a) and ADC (Figure 5b) MRI images from 17 ischemic stroke patients and estimated infarct volumes (Figure 5c). Median infarct volumes were 511 mm^3^ (316–1057) (Figure 5c).

As the inflammatory process that ensues after a stroke destabilizes the BBB and contributes to neuro-axonal damage, resulting in the release of neurofilaments and glial markers into the blood [51,52,53], we assessed serum NF-L and GFAP levels (Figure 5d–e). We found that serum NF-L levels were significantly increased in ischemic stroke patients [17.20 pg/mL (9.65; 58.00)] compared to healthy controls [8.05 pg/mL (6.20; 13.18), U = 9.50, *p* = 0.013]. In addition, serum GFAP levels were significantly increased in ischemic stroke patients [152.0 pg/mL (95.5; 297.5)] compared to healthy controls [90.5 pg/mL (52.5; 109.0), U = 74, *p* = 0.008)]. Serum NF-L and GFAP levels were found to positively correlate (Spearman’s rho = 0.58, *p* < 0.0001).

### 3.8. Plasma TNFR1 and TNFR2 Levels Correlate with Serum NF-L Levels but Not with Infarct Volume, Stroke Severity, or Functional Outcome

We initially tested for a correlation between time to first blood sample and plasma levels of TNFR1 and TNFR2. Despite a trend for TNFR1, we found no significant association between time to first blood sample and plasma TNFR1 (Spearman’s rho = −0.28, *p* = 0.08) or TNFR2 (Spearman’s rho = −0.25, *p* = 0.13). There was also no correlation between SSS scores and plasma TNFR1 (Spearman’s rho = −0.18, *p* = 0.29) or plasma TNFR2 (Spearman’s rho = −0.20, *p* = 0.24). No correlation was found between IFV and plasma TNFR1 (Spearman’s rho = −0.39, *p* = 0.13) or TNFR2 (Spearman’s rho = −0.27, *p* = 0.32), nor between mRS at three months and TNFR1 (Spearman’s rho = 0.10, *p* = 0.61) or TNFR2 (Spearman’s rho = 0.12, *p* = 0.54). Interestingly, serum NF-L levels correlated with plasma TNFR1 (Spearman’s rho = 0.56, *p* = 0.001) and TNFR2 (Spearman’s rho = 0.65, *p* < 0.0001) levels, and SSS (Spearman’s rho = −0.51, *p* = 0.006), but not with IFV (Spearman’s rho = −0.49, *p* = 0.36) or time to first blood sample (Spearman’s rho = 0.13, *p* = 0.48). In contrast, serum GFAP levels did not correlate with plasma TNFR1 (Spearman’s rho = 0.22, *p* = 0.25) or TNFR2 (Spearman’s rho = 0.12, *p* = 0.54) levels, SSS (Spearman’s rho = −0.29, *p* = 0.14), IFV (Spearman’s rho = −0.77, *p* = 0.10), or time to first blood sample (Spearman’s rho = −0.24, *p* = 0.18).

### 3.9. Plasma IL-6 Levels Were Increased in Ischemic Stroke Patients and Correlated with Plasma TNFR1 and TNFR2 Levels

As VEGF is known to induce endothelial proliferation and increase endothelial permeability contributing to BBB breakdown [38,39], we measured plasma VEGF-A levels (Table 3). However, we observed no significant difference in plasma VEGF-A between ischemic stroke patients and healthy controls (*p* = 0.84). VEGF-A levels did not show any correlation to TNF (Spearman’s rho = −0.19, *p* = 0.31), TNFR1 (Spearman’s rho = 0.02, *p* = 0.93), or TNFR2 (Spearman’s rho = −0.05, *p* = 0.80).

As plasma IL-6 levels obtained within the first week post-stroke have been shown to correlate with brain infarct volume, stroke severity, and long-term outcome [12,54,55,56], we also investigated plasma IL-6 levels and their possible correlation with TNF, TNFR1, and TNFR2. We found that plasma IL-6 levels were significantly increased in ischemic stroke patients compared to healthy controls (*p* = 0.04) (Table 3), and were positively correlated to both plasma TNFR1 (Spearman’s rho = 0.41, *p* = 0.02) and TNFR2 levels (Spearman’s rho = 0.38, *p* = 0.03), although not to plasma TNF levels (Spearman’s rho = 0.21, *p* = 0.24).

## 4. Discussion

In the present study, we found that plasma TNFR1 and TNFR2 levels were increased in the acute phase in ischemic stroke patients. This is in line with findings of a previous study by our lab, where plasma levels of both receptors were increased in blood samples taken at an average of 8 h post ischemic stroke, but not at 72 h [3]. A previous study of ischemic stroke found increased plasma TNFR1 at 5–7 days after ischemia but not at other timepoints, whereas TNFR2 was not elevated [14]. Blood samples in the current study were taken at an average of 24.4 h post-stroke, suggesting that TNFR1 and TNFR2 levels are influential in both the acute and subacute stages of stroke.

The increased plasma TNFR1 and TNFR2 levels correlated strongly with each other, implying that the two receptors respond in parallel, and this knowledge could contribute to locating the origin of the response. High post-stroke TNFR1 levels are associated with a poor outcome and increase the risk of secondary vascular events [15]. While TNFR1 is expressed in most tissues, TNFR2 is mainly expressed on monocytes and neutrophils (https://www.proteinatlas.org/). This suggests that an inflammatory response leading to secretion of TNFR1 and TNFR2 from these cells may contribute to the rise in plasma TNFR1 and TNFR2 levels after stroke. TNFR1 and TNFR2 are also expressed on endothelial cells in the brain [17], which could involve vascular damage and leakage cross the BBB [57]. Interestingly, in post-mortem brain tissue derived from ischemic stroke patients, besides macrophage expression, TNFR1 expression also increases on neurons and glial cells, whereas TNFR2 increases mainly on astrocytes [3,47]. This increased expression appeared to take place already within the first two days post-stroke. The findings of increased NF-L and GFAP levels in the blood of ischemic stroke patients suggest that brain-derived proteins leak to the periphery quickly after the ischemic event. It is therefore plausible that the increased levels of soluble TNFR1 and TNFR2 observed in the present study can be derived from brain-resident cells. This is supported by our findings of a correlation between TNFR1 or TNFR2 and NF-L levels, and, although we did not find a correlation between TNFR1 or TNFR2 and GFAP, the increase in serum GFAP in ischemic stroke patients compared to controls similarly demonstrates that brain proteins leak to the periphery post-stroke.

Classical monocytes residing in the bone marrow are released into the circulation where they are present for 24 h before mostly entering tissues; 1% of them differentiate into intermediate monocytes further differentiating into non-classical monocytes [35]. The kinetics of monocytes is tightly regulated in steady state situations, but stress can lead to rapid release of classical monocytes into the circulation and thus alter the distribution of monocyte subsets [35]. Urra et al. found increased proportion of classical monocytes to inversely relate to infarct size in stroke [36], suggesting that bone marrow release of classical monocytes may be initiated by stroke. Cerebral ischemia in mice leads to a rapid increase in Ly6C^high^ monocytes in the blood due to release from the bone marrow and then a decrease after 24 h [58]. Kaito and colleagues found an increased percentage of classical monocytes 0–7 days after ischemia and of intermediate monocytes 3–19 days after ischemia [59]. In the current study, we did not see an increase of classical monocytes in the peripheral blood at average 24 h post-stroke, possible due to the late time point (when classical monocytes may already have been recruited into the CNS) or the variability of sampling times. Investigation of monocyte profiling at several time points post-stroke is essential to further explore the dynamics and release of monocytes after human ischemic injury.

We found that TNFR1 was expressed on all monocyte subsets and on neutrophils, with the highest percentage on classical monocytes. This is in accordance with other studies [60,61,62]. The percentages of TNFR1-expressing non-classical monocytes and neutrophils were significantly lower after ischemia, which may reflect changes in expression or cleaving of the membrane-bound TNFR1. TNFR2 was expressed on most non-classical monocytes, about half of the intermediate monocytes, and on approximately 10% of classical monocytes. In response to ischemia, we found a trend towards increased TNFR2-expressing non-classical and intermediate monocyte populations and a higher MFI expression of TNFR2 on intermediate monocytes. A study comparing expression of TNFR1 and TNFR2 in controls and patients with sarcoidosis found significant cell-specific differences in expression levels [60]. In our study, neutrophils had a high expression of TNFR2 which was enhanced following ischemia. Monocyte expression of TNFR1 has been found to increase cell survival in mice models and subsequently alter the distribution of monocyte subsets [35]. The inflammatory properties of monocytes are influenced by their ability to respond to TNF through TNFR1 and TNFR2 [35]. TNF can also induce survival in neutrophils through nuclear factor kappa-B mediated signaling [63]. In mice studies, TNFR1 has been shown to be important for post-ischemic angiogenesis [64]. While TNFR1 and TNFR2 can be upregulated in response to stimulation with lipopolysaccharide [65], the mechanisms leading to regulation following stroke remain unclear.

Both receptors can shed to become soluble forms, perhaps also indicating that peripheral immune cells to contribute to the inflammatory response, as soluble TNF receptors can function as natural inhibitors of TNF.

In the present study, we also found CCL2 and CCR2 levels to be significantly increased in the blood of our ischemic stroke patients. Furthermore, we found CCR2 but not CCL2 to correlate with time to blood sample, SSS, and mRS, suggesting that CCR2 may be a potential biomarker for stroke severity and functional outcome. However, whether this is the case awaits further investigations.

CCL2 has previously been identified as a marker for secondary brain ischemia in mice, and it is suggested to be a good therapeutic target in ischemia [34]. CCR2 is highly expressed on classical monocytes and promotes their migration from bone marrow to the periphery [66]. The Ly6C^high^ murine equivalent of the human classical monocytes and neutrophils enters ischemic lesions in the brain in a CCR2-dependent manner [67,68], and the ablation of CCR2 decreases infarct size [34], although CCR2 monocytes have also been shown to be neuroprotective after stroke [69]. We found the highest CCR2 frequency and expression levels on classical monocytes, followed by intermediate monocyte whereas the non-classical monocytes had almost no expression of CCR2, which is in accordance with previously published data [70]. We found comparable CCR2 expression patterns on monocytes and neutrophils in healthy controls and ischemic stroke patients, suggesting that CCR2 expression on peripheral monocytes and neutrophils is not regulated in response to ischemia. We did not measure the level of CCL2 specifically in leukocytes, and it would be preferable to do so in order to establish the CCL2/CCR2 axis as a target of interest in humans. CCR2 has a crucial function in scavenger of CCL2 from the blood [71] and a dysfunction in CCR2 is associated with early ischemic heart disease [72]. Whether CCR2 released into the plasma may also render a protective function in inhibiting the CCL2 effect and protecting against clotting of platelets and activation on monocytes is not known [73]. CCR2 is expressed on a subset of neutrophils and is regulated by immune activation enabling the neutrophils to invade the brain after ischemic stroke [67]. In our study, the neutrophils showed a trend towards increased CCR2 expression in ischemic stroke. Others have found that neutrophils can upregulate CCR2 expression in response to inflammatory mediators [74], and it remains to be investigated if this activation of neutrophils also takes place following ischemic stroke. In mice, the invasion of non-classical monocytes at sub-acute timepoints recruits neutrophils, thereby contributing to secondary brain damage [75]. Although we found a trend towards upregulation of TNFR1 in classical monocytes and of TNFR2 in intermediate and non-classical monocytes and neutrophils, this was not correlated with CCR2 expression as the TNFR1 and TNFR2 expression was comparable in all CCR2^+^ cells.

The subdivision of human monocytes into classical, non-classical, and intermediate is based on the expression levels of the lipopolysaccharide coreceptor CD14 and the Fcγ receptor III CD16. The distribution of classical, intermediate, and non-classical monocytes in human peripheral blood is tightly regulated and remains stable over time [76]. The monocyte subsets have distinct surface receptor expressions and differentiated cytokine profiles, where the classical monocyte is considered pro-inflammatory and produces higher amounts of TNF, IL-1β, and IL-6 in response to stimulation [76]. We found increased levels of IL-6 post-stroke, pointing towards an activation of classical monocytes. This is in agreement with a study of cerebral ischemia in neonates, where early response to ischemia led to an increase of IL-6 [77]. The classical monocyte readily enters inflamed tissue and is believed to be the main subset of bone marrow-derived monocytes entering the CNS after ischemic stroke [36,78].

To assess the consequence of increased plasma TNFR1 and TNFR2 levels after ischemic stroke, we evaluated their association with infarct volume but found no correlation. This is in line with a previous clinical study in which plasma levels of TNFR1 and TNFR2 in patients of intracerebral hemorrhage (ICH) were not associated with the development of larger infarct volumes [17]. Assuming that TNF receptors in the plasma originate from peripheral monocytes, a mouse study showed that removal of the spleen reduced the number of monocytes present in the infarct after stroke, but no correlation to the infarct volume was found [58]. This contrasts with a preclinical study showing that injection of soluble TNFR1 in rats 6 h after experimentally induced stroke reduced the infarct volume [79]. The role of soluble TNF receptors on infarct development is thus unclear.

To investigate if elevated TNF receptor levels could have prognostic value, we investigated a possible association with the patients’ mRS scores but found no statistical significance for either receptor. This is in contrast to Svensson and colleagues, who showed a correlation to both TNFR1 and TNFR2 in ICH patients [17]. Our lack of correlation between TNFR expression and functional recovery may have been due to the study cohort consisting of patients with minor to moderate stroke. TNFR1 showed a trend towards an upregulation post-stroke in classical and intermediate monocytes, the final differential states of non-classical monocytes. This may affect the survival time and pro-inflammatory cytokine profile of the monocytes in ischemic stroke patients.

## 5. Conclusions

We found that both TNFR1 and TNFR2 plasma levels increased in ischemic stroke patients but showed no correlation with patient outcome measurements. Compared to healthy controls, non-classical monocyte and neutrophil populations expressing TNFR1 decreased and non-classical populations co-expressing both TNFR1 and TNFR2 decreased. Neutrophil populations expressing TNFR2 increased in ischemic stroke patients compared to healthy controls, as did TNFR2 expression on intermediate monocytes. This study supports the hypothesis of a response orchestrated by the peripheral immune system following an ischemic stroke. However, the origin of the increased TNFR1 and TNFR2 plasma levels could not be clearly linked to peripheral monocytes or neutrophils. Further studies are recommended in this field to clarify the potential role as treatment target.

## Figures and Tables

**Table 1:** Characteristics of study participants. In two cases of wake-up stroke, the time of first symptom was unknown, and the time for wake-up was used (10.9 and 32.8 h, respectively).

	Controls	Ischemic Stroke	*p*-Value	^b^ Ischemic Stroke
Number of participants	10	33		11
Age, years, median (IQR)	59.0 (54.8; 72.3)	73.0 (62.5; 80.5)	0.02 ^c^	62.0 (48.0; 76.0)
Sex, *n* (%) males	2 (20)	20 (60.6)	0.03 ^d^	7 (63.6)
Anti-inflammatory medication, *n* (%) - Yes - No	1 (10) 9 (90)	10 (30.3) 23 (69.7)	0.41 ^d^	3 (27.3) 8 (72.7)
Smoking, *n* (%)				
- Current smoker - Previous smoker - Never smoker - Not known	1 (10) 5 (50) 4 (40) 0 (0)	8 (24.2) 9 (27.3) 10 (30.3) 6 (18.2)	0.26 ^e^	2 (18.2) 4 (36.4) 2 (18.2) 3 (27.2)
Alcohol consumption ^a^, *n* (%)				
- <Max recommended levels - >Max recommended levels - Not known	10 (100) 0 (0) 0 (0)	23 (69.7) 1 (3) 9 (27.3)	0.14 ^e^	3 (27.3) 7 (63.6) 1 (9.1)
SSS score (median + IQR)		52.0 (43.5; 56.0) (5 missing)		52.0 (49.3; 57.3) (1 missing)
mRS score (median + IQR)		0.0 (0.0; 1.0) (14 missing)		1.0 (0.5; 1.5) (2 missing)
Treatment, *n* (%)				
- Thrombolysis - Thrombectomy - None		12 (36.4) 1 (3) 20 (60.6)		0 (0) 0 (0) 11 (100)
Time to blood sample, hours (median + IQR)		23.2 (15.1; 32.0)		10.8 (8.4; 15.58)
Sodium, mmol/L (median + IQR)		139.0 (136.5; 141.0)		139.5 (136.8; 141.0)
Potassium, mmol/L (median + IQR)		4.0 (3.9; 4.2)		3.8 (3.7; 4.1)
CRP, mg/L (median + IQR)		2.95 (1.23; 6.88)		2.55 (0.95; 4.05)

mRS, modified Rankin scale; NSAID, non-steroidal anti-inflammatory drug; SSS, Scandinavian Stroke Scale. ^a^ The Danish Health authorities recommend maximal <7 units per week for women and maximal <14 units per week for men (1 unit equals one glass of wine); ^b^ Data obtained from Nielsen et al. [40] and Clausen et al. [3]; ^c^ Mann–Whitney test; ^d^ Fisher’s exact test; ^e^ Chi-square test.

**Table 2:** Differential leukocyte counts of ischemic stroke patients. As differential leukocyte counts were not available for healthy controls, reference ranges for a normal population are given as comparison.

Differential Leukocyte Count on Admission ^a^	Mean	Median (IQR)	2.5–97.5% PCTL	Reference Range of Normal Population 2.5–97.5% PCTL ^b^
Total leukocyte count (*n* = 33)	8.9	7.6 (6.1; 9.6)	2.4–38.5	3.5–8.8
Thrombocyte count				
- Men, *n* = 20 - Women, *n* = 13	222.3 284.7	230.0 (158.0; 264.5) 281.0 (205.5; 336.0)	130.0–336.0 182.0–418.0	Men: 145–350 Women: 165–400
Neutrophil count (*n* = 33)	5.6	5.1 (3.6; 6.6)	1.7–13.1	1.5–7.5
Lymphocyte count (*n* = 31)	2.5	1.6 (1.2; 1.9)	0.6–31.5	1.0–4.0
Monocyte count (*n* = 31)	0.7	0.6 (0.5; 0.7)	0.3–1.6	0.2–0.8

For the study cohort, IQR (25–75%) and PCTL (2.5–97.5%) are provided. ^a^ All values are given in cell count ×10^9^/L whole blood. N, number of participants; IQR, interquartile range; PCTL, percentile. ^b^ Reference range expresses 2.5–97.5% PCTL.

**Table 3:** Plasma cytokine, receptor, and growth factor levels. Data are presented as median (IQR). CV, coefficient of variation; IL-6, interleukin-6; TNF, tumor necrosis factor; TNFR1, tumor necrosis factor 1; TNFR2, tumor necrosis factor 2; VEGF-A, vascular endothelial growth factor-A; *U*, Mann–Whitney *U* values.

Protein (pg/mL)	Controls (*n* = 10)	Ischemic Stroke (*n* = 33)	*U*	*p*-Value	Mean CV (%)
TNF	2.1 (1.8; 2.5)	2.2 (1.9–2.9)	141	0.50	5.9
TNFR1	306.5 (257.4; 366.5)	395.1 (330.4; 494.5)	93	0.04	3.8
TNFR2	615.4 (561.4; 704.3)	845.7 (675.3; 1156.0)	90	0.03	4.1
IL-6	0.76 (0.41; 1.13)	1.25 (0.65; 2.33)	95	0.04	1.4
VEGF-A	29.1 (22.0; 39.6)	30.2 (20.4; 38.6)	162	0.84	8.9

**Table 4:** TNFR1 and TNFR2 expression on CCR2^+^ leukocytes. Data are presented as median (25–75% IQR). *U*, Mann–Whitney *U* values.

Cell Population	Controls (*n* = 8)	Ischemic Stroke (*n* = 23)	*U*	*p*-Value
TNFR1^+^ CCR2^+^ classical monocytes	2.29% (1.35–8.06)	2.77% (1.80–5.05)	89	0.91
TNFR1^+^ CCR2^+^ non-classical monocytes	4.42% (0.97–6.09)	1.83% (0.33–4.82)	68	0.21
TNFR1^+^ CCR2^+^ intermediate monocytes	1.50% (0.42–1.94)	0.38% (0.00–0.84)	55.5	0.10
TNFR1^+^ CCR2^+^ neutrophils	1.63% (0.55–4.53)	2.56% (0.18–9.51)	78	0.55
TNFR2^+^ CCR2^+^ classical monocytes	12.59% (3.65–35.02)	9.37% (4.92–30.35)	89.5	0.92
TNFR2^+^ CCR2^+^ non-classical monocytes	46.92% (32.01–60.51)	40.65% (27.48–52.73)	75	0.46
TNFR2^+^ CCR2^+^ intermediate monocytes	34.09% (28.79–51.01)	43.82% (30.47–53.36)	67.5	0.28
TNFR2^+^ CCR2^+^ neutrophils	54.70% (31.19–63.78)	44.87% (31.22–66.21)	82	0.67
TNFR1^+^TNFR2^+^ CCR2^+^ classical monocytes	1.10% (0.67–2.24)	1.04% (0.77–1.61)	88	0.52
TNFR1^+^TNFR2^+^ CCR2^+^ non-classical monocytes	7.92% (1.24–18.39)	10.37% (0.54–19.32)	88	0.88
TNFR1^+^TNFR2^+^ CCR2^+^ intermediate monocytes	3.88% (2.72–9.66)	5.76% (2.51–18.41)	75	0.87
TNFR1^+^TNFR2^+^ CCR2^+^ neutrophils	6.05% (5.00–21.29)	20.36% (3.68–30.21)	77	0.46

## Data Availability

All data are hosted at Open Patient data Explorative Network (OPEN; https://open.rsyd.dk/) and requests to access datasets should be directed to klam-bertsen@health.sdu.dk.

## Supplementary Materials

The following are available online at https://www.mdpi.com/article/10.3390/cells10040861/s1, Table S1. Characteristics of study participants for CCL2 and CCR2 analysis.
