# Supplementary material for: Leukocyte TNFR1 and TNFR2 Expression Contributes to the Peripheral Immune Response in Cases with Ischemic Stroke
Source: Cells. 2021 Apr 9;10(4):861. doi: 10.3390/cells10040861 (PMC8069317; doi:10.3390/cells10040861)
Supplement: Supplementary file 1 [file cells-10-00861-s001.pdf]

cells

MDPI

Article

Leukocyte TNFR1 and TNFR2 expression contributes to the peripheral immune response in cases with ischemic stroke

Rikke Birk Hansen [1,2], Cathrine C.H. Laursen [1,3], Niala Nawaz [1,2], Jonna S. Madsen[4,5], Helle H. Nielsen [1,2,3], Christina Kruuse [6,7], Arne Møller [8,9], Matilda Degn [10]* and Kate Lykke Lambertsen [1,2,3,11]*

1 Department of Neurobiology Research, Institute of Molecular Medicine, University of Southern Denmark, 5000 Odense, Denmark; rikkebirkhansen@gmail.com (R.B.H.); laursencathrine@gmail.com (C.C.H.L.); ninaw19@student.sdu.dk (N.N.); Helle.Hvilsted.Nielsen@rsyd.dk (H.H.N.); klambertsen@health.sdu.dk (K.L.L.)
2 Department of Neurology, Odense University Hospital, 5000 Odense, Denmark
3 BRIDGE, Brain Research - Inter-Disciplinary Guided Excellence, Department of Clinical Research, University of Southern Denmark, 5000 Odense, Denmark
4 Department of Biochemistry and Immunology, Lillebaelt Hospital, University Hospital of Southern Denmark, 7100 Vejle, Denmark; Jonna.Skov.Madsen@rsyd.dk
5 Department of Regional Health Research, University of Southern Denmark, 5000 Odense, Denmark
6 Department of Clinical Medicine, University of Copenhagen, 2100 Copenhagen, Denmark; christina.kruuse@regionh.dk (C.K.)
7 Department of Neurology, Herlev Gentofte Hospital, 2730 Herlev, Denmark
8 Department of Nuclear Medicine and PET Center, Aarhus University Hospital, 8200 Aarhus, Denmark; arne@cfin.au.dk (A.M.)
9 Institute of Clinical Medicine, Center of Functionally Integrative Neuroscience, 8000 Aarhus, Denmark
10 Pediatric Oncology Laboratory, Department of Pediatrics and Adolescent Medicine, University Hospital Rigshospitalet, 2100 Copenhagen, Denmark; matildadegn@gmail.com (M.D.)
11 OPEN – Open Patient data Explorative Network, Odense University Hospital, Department of Clinical Research, University of Southern Denmark, 5000 Odense, Denmark
* Correspondence: matildadegn@gmail.com (M.D.) and klambertsen@health.sdu.dk (K.L.L.); Tel.: +45-6550-3806 (K.L.L.) and +45-6061-0084 (M.D.)

Scheme 1. Characteristics of study participants for CCL2 and CCR2 analysis. mRS, modified Rankin scale; NSAID, non-steroidal anti-inflammatory drug; SSS, Scandinavian Stroke Scale. [§]The Danish Health authorities recommend <7 units per week for women and <14 units per week for men (1 unit equals one glass of wine). [a]Mann Whitney test, [b]Fisher's exact test, [c]Chi-square test.

	Controls	Ischemic Stroke	*p*-Value
**Number of participants**	17	59	
**Age, years, median (IQR)**	57 (49;64)	69 (68;76)	0.003[a]
**Sex, n (%) males**	6 (35)	36 (61)	0.09[b]
**Anti-inflammatory medication, n (%)**			
- Yes	2 (12)	20 (34)	0.12[b]
- No	15 (88)	39 (66)	
**Smoking, n (%)**			
- Current smoker	2 (12)	13(22)	
- Previous smoker	9(53)	23 (39)	0.16[c]
- Never smoker	6(35)	13(22)	
- Not known	0(0)	10 (17)	
**Alcohol consumption[a], n (%)**			
- < Recommended levels	16 (100)	43 (73)	0.13[c]
- > Recommended levels	1 (0)	5 (8)	
- Not known	0 (0)	11 (19)	
**SSS score (median + IQR)**		52.0 (49;54) (9 missing)	
**mRS score (median + IQR)**		1.1 (0.6; 1.6) (29 missing)	
**Treatment, n (%)**			
- Thrombolysis		16(27)	
- Thrombectomy		3(5)	
- None		36(61)	
**Time to blood sample, minutes (median + IQR)**		925 (643;1165)	

Publisher's Note: MDPI stays neutral with regard to jurisdictional claims in published maps and institutional affiliations.
